# Oral anticoagulant use and the development of new cerebral microbleeds in cardioembolic stroke patients with atrial fibrillation

**DOI:** 10.1371/journal.pone.0238456

**Published:** 2020-09-17

**Authors:** Toshitaka Umemura, Shinichi Mashita, Takahiko Kawamura

**Affiliations:** 1 Department of Neurology, Chubu Rosai Hospital, Japan Organization of Occupational Health and Safety, Nagoya, Japan; 2 Department of Radiology, Chubu Rosai Hospital, Japan Organization of Occupational Health and Safety Nagoya, Japan; 3 Department of Diabetes and Endocrine Internal Medicine, Preventive Medical Center, Chubu Rosai Hospital, Japan Organization of Occupational Health and Safety Nagoya, Nagoya, Japan; Institut d'Investigacions Biomediques de Barcelona, SPAIN

## Abstract

**Objective:**

Cerebral microbleeds (CMBs) are a magnetic resonance imaging (MRI) marker for cerebral small vessel disease. Existing CMBs and those that newly develop are associated with the risks of stroke incidence and recurrence. The purpose of the present study was to investigate the association of oral anticoagulant (OAC) use and the development of new CMBs in cardioembolic stroke patients with atrial fibrillation.

**Subjects and methods:**

We prospectively followed cardioembolic stroke patients with atrial fibrillation who had been hospitalized in the stroke center of our hospital, had been prescribed anticoagulants at discharge, and underwent repeated brain MRI with an interval of at least one year from the baseline MRI. Assessing the presence, number and location of CMBs using T2*-weighted gradient-recalled echo MRI, we used logistic regression models to investigate the associations between OAC use and the incidence of new CMBs. We also examined associations of subsequent stroke with OACs and CMBs during the follow-up.

**Results:**

A total of 81 patients, consisting of 45 patients receiving direct oral anticoagulants (DOACs) and 36 patients receiving warfarin (WF), were analyzed in the present study. Baseline CMBs were observed in 19/81 patients (23.5%) and new CMBs in 18/81 patients (22.2%) on follow-up MRI (median interval, 34 months). Of the 31 new CMBs, 25 (80.6%) developed in the lobar location and 6 (19.4%) in the deep or infratentorial location. New CMBs occurred in 4 patients (10.0%) taking DOACs　alone, in 10 patients (35.7%) taking WF alone, in 3 patients (37.5%) taking WF plus antiplatelet agents and in 1 patient (20.0%) taking DOAC plus antiplatelet agent. Regarding location, the new CMBs were the lobar type in 7 of the 10 patients taking WF alone, as well as in 3 of the 4 patients taking DOACs alone. In multivariate analysis, the presence of CMBs at baseline and WF use (vs. DOAC use) were associated with new CMBs (CMB presence at baseline: OR 4.16, 95% CI 1.19–14.44; WF use: OR 3.38, 95% CI 1.02–11.42). The presence of ≥ 2 CMBs at baseline was related to a higher risk of subsequent stroke (OR 7.25, 95% CI 1.01–52.35, P = 0.048).

**Conclusion:**

Our findings suggest that DOAC compared with WF use at discharge is associated with a lower incidence of new CMBs in cardioembolic stroke patients with atrial fibrillation. Further prospective studies in the clinical setting are needed to confirm our exploratory data.

## Introduction

Histopathologically, cerebral microbleeds (CMBs) appear as clusters of hemosiderin-containing macrophage with a pericapillary location [1,2] and as small, rounded, hypointense lesions on T2*-weighted gradient-recalled echo magnetic resonance imaging (MRI). Deep CMBs are thought to be associated with hypertensive vasculopathy and lobar CMBs with cerebral amyloid angiopathy (CAA), but the findings of many studies suggest that CMB-related factors differ [3–6]. CMBs are neuroimaging markers of cerebral small vessel disease and the association of their presence with risk of stroke development or recurrence is clinically important [7–9].

While previous research demonstrated that lobar CMBs increased the risk of intracerebral hemorrhage accompanying warfarin (WF) use [10], in clinical studies, direct oral anticoagulants (DOACs) markedly reduced the risk of intracerebral hemorrhage compared with WF [11,12]. In daily clinical practice, although DOAC usage for prevention of stroke onset and recurrence in patients with atrial fibrillation is increasing, few studies have longitudinally examined the association between DOAC use and the incidence of CMBs. The main objective of this study was to observe T2*-weighted MRI in cardioembolic stroke patients with atrial fibrillation and longitudinally assess an association of OAC use and new CMBs. We also investigated associations of subsequent stroke that occurred during the follow-up period with OAC use and CMB burden.

## Subjects and methods

### Patients

**Fig 1:**
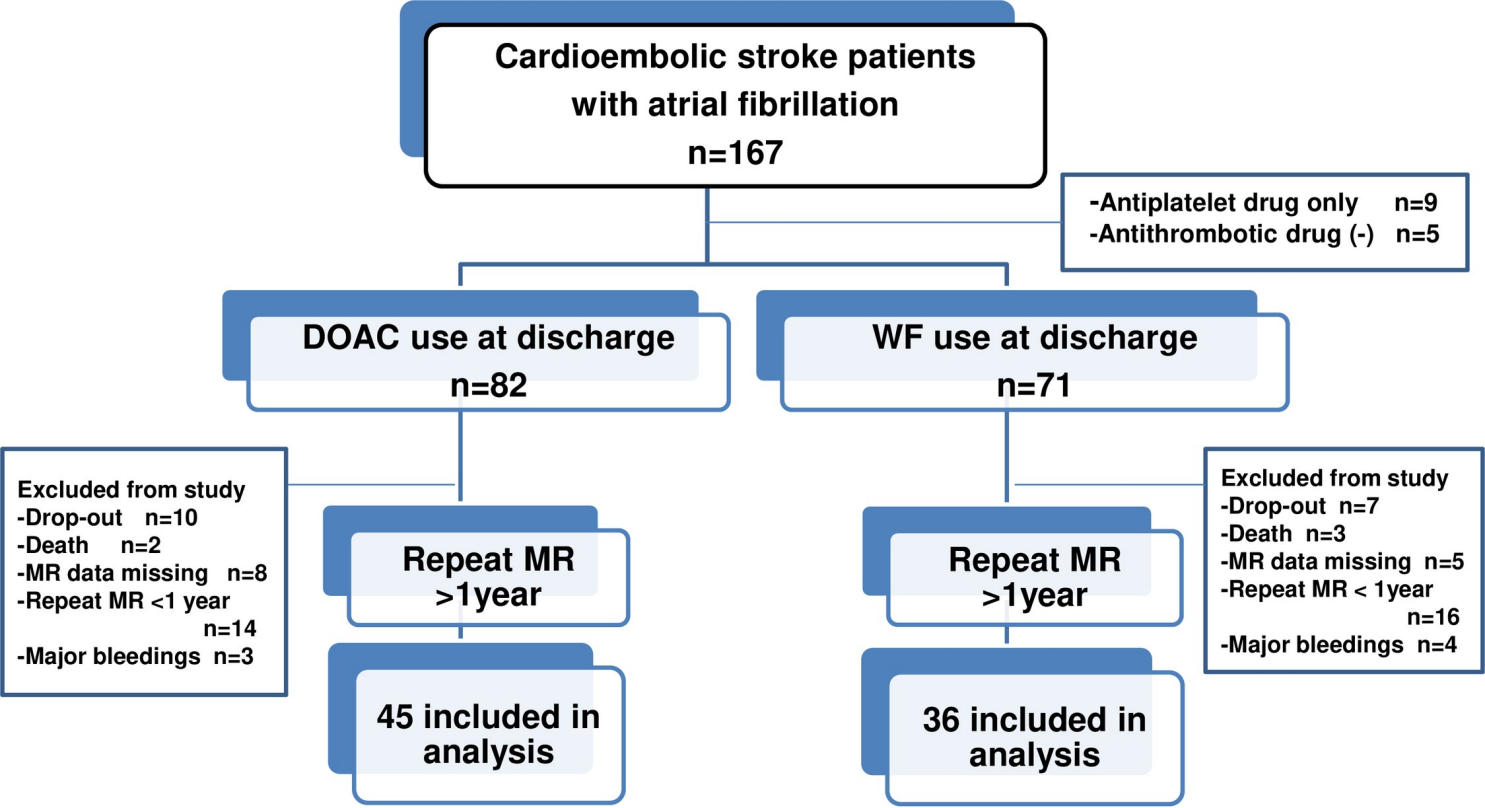
Flow diagram of the present study. Patients who had been prescribed anticoagulants at discharge and were able to visit our hospital as outpatients were included in the present study.

We prospectively followed cardioembolic stroke patients with atrial fibrillation who had been hospitalized in the stroke center of our hospital, had been prescribed OACs (alone or concomitant with antiplatelet agents) at discharge, and were able to visit the hospital as outpatients. The present study enrolled 153 patients for evaluation of clinical data, laboratory tests and baseline and follow-up MRI. Patients were excluded if any of the following applied: presence of pacemaker or prosthetic heart valve, undergoing dialysis. Nine patients taking antiplatelet agents only at discharge were excluded from the analysis. Eighty-one patients who underwent repeated brain MRI at our hospital with an interval of at least one year from the baseline MRI were included in the final analysis. As a large number of those enrolled had mild neurological symptoms and therefore could be treated as outpatients, many were referred to a local clinic, accounting for the high drop-out rate. Details of the study sample selection are provided in **Fig 1**. While the choice of oral anticoagulation agent was left to the attending physician, many patients with reduced renal function (eGFR <45 mL/min/1.73 m^2^) and low bodyweight received WF. It was also prescribed for patients with moderate valvular heart disease (n = 7). The observation period of this study was from April 2012 to December 2018. The present study was approved by the ethics committee of Chubu Rosai Hospital. After informed consent was obtained from each of the participants, this study was performed in accordance with the principles of the Declaration of Helsinki.

### Evaluation of stroke risk factors

Hypertension was defined as previously diagnosed and undergoing therapy with antihypertensive agents at the time of admission or both blood pressures exceeding 140/90 mmHg for 3 measurements. Diabetes was defined as currently undergoing diabetic therapy or fasting blood glucose ≥126 mg/dL or HbA1c ≥6.5% (NGSP). Dyslipidemia was defined as either a serum low-density lipoprotein-cholesterol ≥140 mg/dL, high-density lipoprotein-cholesterol <40 mg/dL, triglycerides ≥150 mg/dL, or a history of taking lipid-lowering drugs. Chronic kidney disease (CKD) was defined as the presence of albuminuria (UACR ≥30 mg/g creatinine) and/or low eGFR (<60 mL/min/1.73 m^2^). During the observation period, drug therapy for these risk factors was performed based on the Japanese guidelines for the management of stroke by attending physicians. Smoking status was defined as current use. Atrial fibrillation was defined as recorded history, or recorded by ECG monitor or Holter ECG during hospitalization. The CHADS_2_ score [13] was recorded to estimate stroke risk at the beginning of the observation period. To obtain the CHADS_2_ score, the numbers of points for the present conditions were added together, with 2 points for a history of stroke or transient ischemic attack (TIA) and 1 point for age ≥75 years, hypertension, diabetes or congestive heart failure. The CHA_2_DS_2_-VASc score [14] (2 points for a history of stroke or age ≥75 years and 1 point each for age of 65 to 74 years, a history of hypertension, diabetes, cardiac failure, and vascular disease) was also calculated. TIA was defined as a transient episode of neurological deficit caused by focal brain ischemia without acute infarction.

### Brain MRI acquisition

All of the MRI examinations were performed on a Signa Horizon 1.5T instrument (GE Healthcare, Milwaukee, WI, USA). The MRI protocol consisted of T1-weighted spin echo (inversion recovery: repetition time/echo time (TR/TE = 2380/27.4 ms), T2-weighted fast spin-echo (TR/TE = 4017/103ms), fluid-attenuated inversion-recovery (TR/TE = 8002/146 ms), T2*-weighted gradient-recalled echo (TR/TE = 500/15 ms, flip angle 20°), and diffusion-weighted image (TR/TE = 10000/70 ms; b values of 0 and 1000 s/mm^2^) sequences in the axial plane with a slice thickness of 5 mm and an interslice gap of 2mm [15].

### CMB evaluation

**Fig 2:**
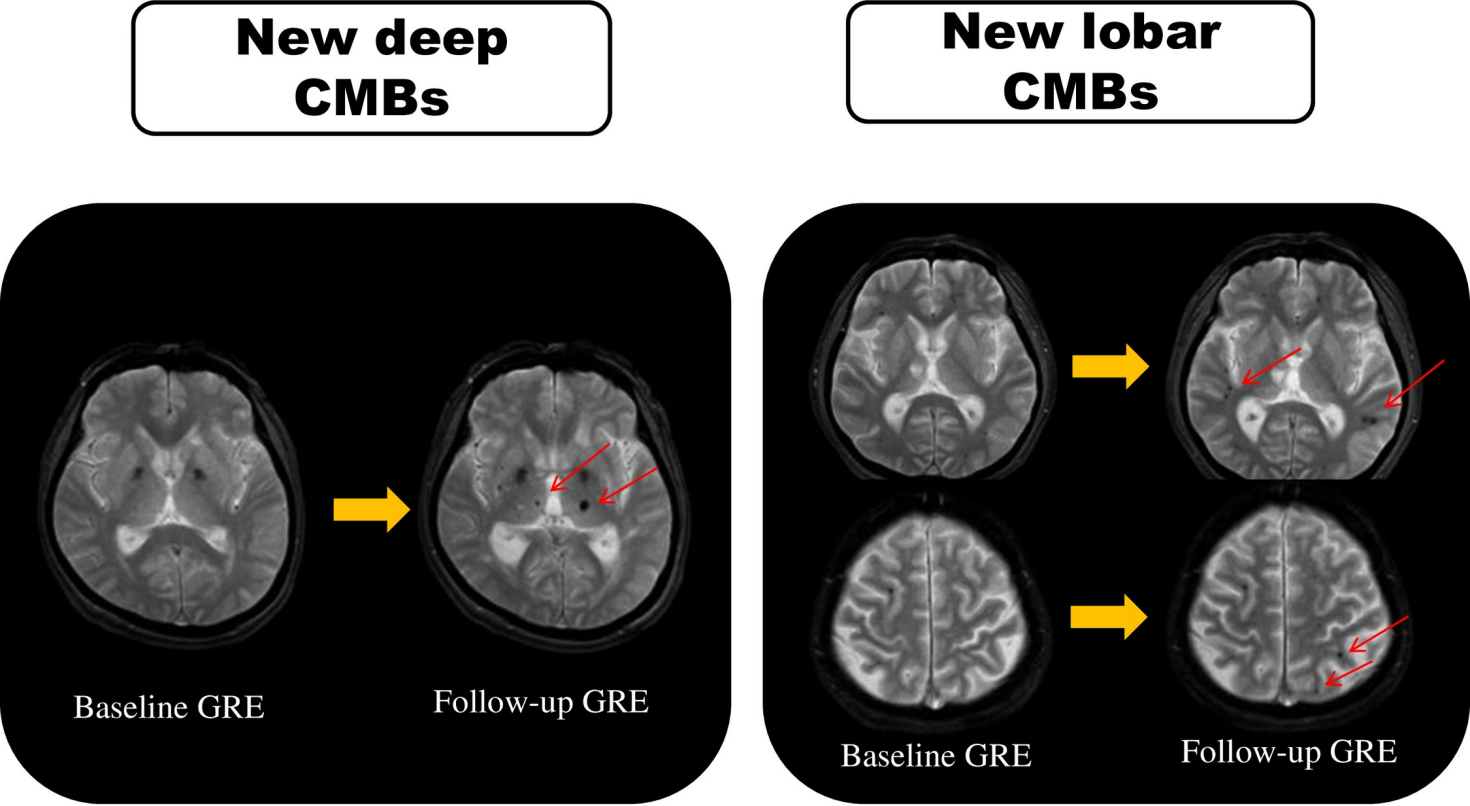
An illustration of MRI in the two patients who showed different types of new CMB locations. For each patient, the baseline GRE is on the left, and the follow-up GRE is on the right. Arrows indicate new CMBs on the follow-up scan. CMBs: cerebral microbleeds. GRE: T2*-weighted gradient-recalled echo imaging.

CMBs were defined as punctate or round hypointensities of 2 to 10 mm in diameter on T2*-weighted images. Using the rating scale proposed by Cordonnier et al. [16], we evaluated CMBs according to the location and number. CMBs were divided by location into three categories as follows: lobar type (cortex, subcortical white matter), deep type (basal ganglia, thalamus, internal or external capsule) and infratentorial type (brain stem, cerebellum) [15,16]. Symmetrical hypointensities in the globus pallidum, which most likely represented calcification or iron deposition, and flow void artifacts of pial blood vessels were disregarded. In a comparison between baseline and final follow-up MRI, if there had been an increase in deep, infratentorial, or lobar CMBs, this was defined as the presence of new CMBs. **Fig 2** is an example of repeat MRI showing different types of new CMBs by location. A trained radiologist who was blinded to laboratory and clinical information assessed images for the presence, location and number of CMBs.

### Assessment of silent lacunar infarcts and white matter lesions

The number of asymptomatic silent lacunar infarcts (SLIs) and the severity of white matter lesions (WMLs) on MRI findings were also examined. We defined SLIs as focal hyperintense areas that were larger than 3 mm in diameter on T2-weighted images, hypointense areas on T1-weighted images, and areas of hypointensity surrounded by a hyperintense rim on FLAIR images. Multiple SLIs were defined as more than three lesions. The severity of WMLs was graded separately for periventricular and subcortical areas. Periventricular WMLs were classified as grade 1 to 3 as follows: grade 1 (mild); pencil-thin lining, grade 2 (moderate); smooth halo, grade 3 (severe); large confluence. Subcortical WMLs were classified into the following 3 grades according to the Fazekas scale: grade 1 (mild); punctuate foci, grade 2 (moderate); beginning confluence, grade 3 (severe); diffuse confluence [17,18]. A grade >1 was regarded as advanced periventricular or subcortical WML.

### Statistical analysis

Continuous variables were indicated as the mean ± SD or median ± quartile range. For intergroup comparisons of continuous variables, unpaired t tests or Mann-Whitney U tests were used as required. Categorical variables were compared using the χ^2^ test or Fisher’s exact test as required. CMB locations were examined according to the categories of pure lobar microbleeds, deep or infratentorial microbleeds, and mixed microbleeds (deep or infratentorial location with concomitant lobar microbleeds). To determine independent factors associated with new CMBs, variables with P < 0.1 in univariate analysis were entered into a multivariate logistic regression analysis. We estimated annualized symptomatic intracerebral hemorrhage and recurrent ischemic stroke event rates (percent per year and 95% CI). Cumulative event-free rates for the time to stroke events were estimated by Kaplan-Meier survival analysis with patients stratified by OAC type. A two-sided P value <0.05 was considered statistically significant. All of the statistical analyses were performed using SPSS 21.0 software (IBM SPSS, Chicago, IL, USA) and the R version 3.6.1 for Windows.

## Results

A total of 81 patients, consisting of 45 patients receiving DOACs and 36 patients receiving WF, were analyzed in the present study. The mean age of the patients in the present study was 73.2 ± 8.2 years and there were 44 men (54.3%). The median follow-up period was 34 months. Regarding the types of antithrombotic agents at discharge, 40 patients (49.4%) were prescribed DOACs alone, 28 patients (34.6%) WF alone, 8 patients (9.9%) WF plus antiplatelet agents and 5 patients (6.2%) DOACs plus antiplatelet agents. For the DOACs, 12 patients received dabigatran, 23 patients rivaroxaban, 6 patients apixaban and 4 patients edoxaban. In the current study, the median time from stroke onset to start of OACs was 6 days (IQR3-9). The median PT-INR of patients treated with WF was 2.1 and the anticoagulation control was relatively good (time in therapeutic range >80%).

### Baseline characteristics of patients

The baseline characteristics of patients according to type of OACs are summarized in **Table 1**. There were no significant differences between the group using DOACs and the group using WF regarding age, sex or prevalence of hypertension, diabetes, chronic kidney disease, or prior stroke/TIA. There were also no significant differences in the prevalence of advanced WMLs or multiple SLIs, CHADS_2_ score, CHA_2_DS_2_-VASc score, National Institutes of Health Stroke Scale at admission, timing of initiation of OACs, or CMB prevalence at baseline or follow-up period between the groups. No significant differences in the background characteristics were observed even when the 8 patients in the WF group and the 5 patients in the DOAC group using antiplatelet agents were excluded.

**Table 1:** Baseline characteristics of patients stratified by type of OACs.

	DOAC group (n = 45)	WF group (n = 36)	P value
**Age, years**	72.8±7.7	73.8±9.0	0.608
**Male, n(%)**	25 (55.6%)	19 (52.8%)	0.803
**Hypertension, n(%)**	25 (55.6%)	20 (55.6%)	0.999
**Diabetes, n(%)**	6 (13.3%)	8 (22.2%)	0.293
**Dyslipidemia, n(%)**	11 (24.4%)	7 (19.4%)	0.591
**Prior stroke/TIA**	8 (17.8%)	8 (22.2%)	0.617
**Systolic blood pressure, mmHg**	132.7±15.9	135.5±13.5	0.435
**Serum glucose, mg/dL**	128.4±60.1	139.8±52.9	0.373
**eGFR < 60 ml/min/1.73 m**^**2**^**, n(%)**	20 (44.4%)	15 (41.7%)	0.802
**BNP, pg/mL**	176[97–265]	192[130–430]	0.396
**CHADS**_**2**_ **score**	3[3–4]	4[3–5]	0.070
**CHA**_**2**_**DS**_**2**_**-VASc score**	4[3–6]	5[4–6]	0.266
**NIHSS at admission**	4[2–10]	5[2–9]	0.877
**Baseline CMBs, n(%)**	11 (24.4%)	8 (22.2%)	0.815
**PVH or DWMH > grade 1, n(%)**	8 (17.8%)	12 (33.3%)	0.107
**Multiple SLIs, n(%)**	15(33.3%)	13(36.1%)	0.794
**Antiplatelet drug use, n(%)**	5 (11.1%)	8 (22.2%)	0.176
**Antihypertensive drug use, n(%)**	22 (48.9%)	16 (44.4%)	0.690
**Statin use, n(%)**	17 (37.8%)	8 (22.2%)	0.132
**Time from stroke onset to start of OACs, days**	5[2–9]	6[3–8]	0.993
**Follow-up period, months**	31[17–50]	37[20–59]	0.124

Data are expressed as the mean ±SD or median [25–75%] TIA: transient ischemic attack, eGFR: estimated glomerular filtration rate.

PVH: perivascular hyperintensity, DWMH: diffuse white matter hyperintensity, SLIs: silent lacunar infarcts, BNP: brain natriuretic peptide.

NIHSS: National Institutes of Health Stroke Scale, OACs: oral anticoagulants, CMBs: cerebral microbleeds, DOAC: direct oral anticoagulant, WF: warfarin.

### Prevalence of CMBs by location and number

CMBs were observed in 19/81 patients (23.5%) at baseline. They were the deep or infratentorial type in 6 patients (31.6%), the lobar type in 8 patients (42.1%) and the mixed type in 5 patients (26.3%). The CMB burden at baseline was ≥ 2 CMBs (n = 6), 1 CMB (n = 13), and no CMB (n = 62). The median number of baseline CMBs was 1 (range 1–8). New CMBs occurred in 18/81 patients (22.2%) on follow-up MRI, which were the deep or infratentorial type in 2 patients (11.1%), the lobar type in 12 patients (66.7%) and the mixed type in 4 patients (22.2%). The CMB burden at follow-up was ≥ 2 CMBs (n = 17), 1 CMB (n = 11), and no CMB (n = 53). The median number of new CMBs among these patients was 2 (range 1–3). On follow-up MRI, we observed 31 new CMBs in 18 patients. Of the 31 new CMBs, 25 (80.6%) developed in the lobar location, and 6 (19.4%) developed in the deep or infratentorial location. The rate of the number of new CMBs was approximately 0.62 lesions per year in all patients (number per patient-years of follow-up).

### OACs and distribution of baseline and new CMBs

**Fig 3:**
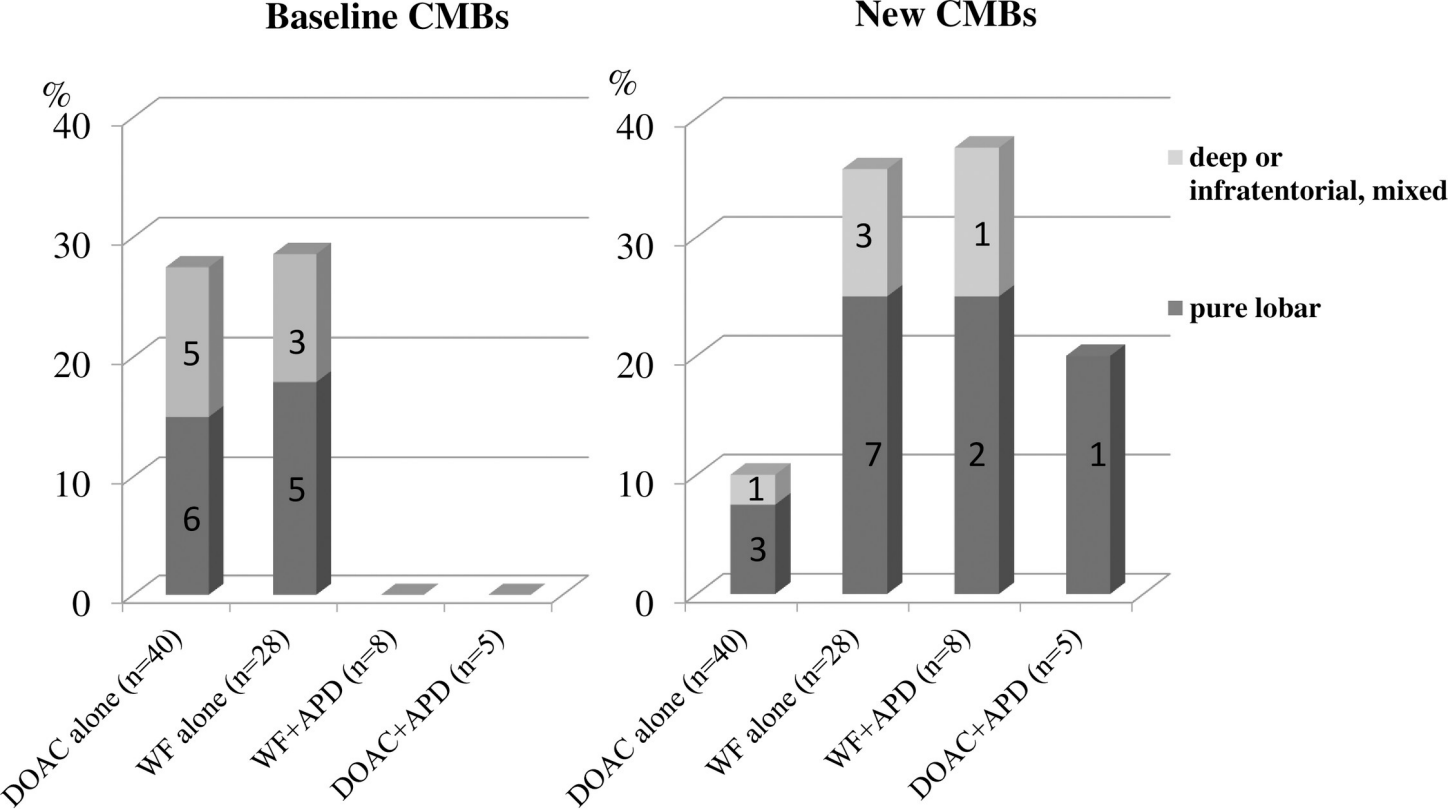
Types of oral anticoagulants and distribution of baseline and new CMBs by location. The bar graph shows the frequency of CMBs stratified by location. Numbers in bars indicate the number of patients with new CMBs stratified by location. * a deep or infratentorial location with concomitant lobar microbleeds. APD: antiplatelet drug.

There were new CMBs in 4 patients (10.0%) taking DOACs alone, in 10 patients (35.7%) taking WF alone, in 3 patients (37.5%) taking WF plus antiplatelet agents and in 1 patient (20.0%) taking DOACs plus antiplatelet agents. New CMBs were significantly more numerous in patients taking WF alone than in those taking DOACs alone (WF 10/28 vs. DOAC 4/40 P <0.01). New CMBs were observed in 4 patients prescribed the Xa inhibitors, but there were none in patients taking the antithrombin agent. Regarding location, the new CMBs were the lobar type in 7 of 10 patients taking WF alone and were the lobar type in 3 of 4 patients taking DOACs alone **(Fig 3).**

### Factors associated with the development of new CMBs

In univariate analysis, advanced WMLs (> Fazekas grade 1), the presence of CMBs at baseline, and WF use (vs. DOAC use) were associated with new CMBs. In multivariate analysis, the presence of CMBs at baseline and WF use (vs. DOAC use) were associated with new CMBs (baseline CMB presence: OR 4.16, 95% CI 1.19–14.44; WF use: OR 3.38, 95% CI 1.02–11.42) **(Table 2).**

**Table 2:** Factors associated with the development of new CMBs.

	Univariate			Multivariate		
Variables	Odds ratio	95% CI	P value	Odds ratio	95% CI	P value
**Age, 1y increase**	1.050	0.982–1.124	0.152			
**Male**	1.066	0.372–3.058	0.905			
**Hypertension**	1.001	0.348–2.870	0.999			
**Diabetes**	2.308	0.661–8.053	0.190			
**eGFR < 60 ml/min/1.73 m**^**2**^	1.900	0.350–5.473	0.234			
**PVH or DWMH > Fazekas grade 1**	3.400	1.107–10.45	0.033	2.088	0.579–7.529	0.261
**Multiple SLIs**	2.720	0.845–9.620	0.126			
**CHADS**_**2**_ **score**	1.669	0.932–2.988	0.085	1.179	0.779–1.785	0.436
**CHA**_**2**_**DS**_**2**_**-VASc score**	1.310	0.917–1.872	0.138			
**Baseline CMBs (+)**	3.782	1.216–11.76	0.022	4.156	1.196–14.44	0.025
**Antiplatelet drug use**	2.644	0.742–9.420	0.134			
**Follow-up period, 1y increase**	1.361	0.814–2.277	0.235			
**WF use vs DOAC use**	3.250	1.077–9.804	0.036	3.383	1.020–11.42	0.046

eGFR: estimated glomerular filtration rate, PVH: perivascular hyperintensity, DWMH: diffuse white matter hyperintensity.

SLIs: silent lacunar infarcts, CMBs: cerebral microbleeds, WF: warfarin, DOAC: direct oral anticoagulant.

### Association between OACs and subsequent stroke during the follow-up period

Subsequent stroke occurred in 9 patients (3.9, 95% CI 1.8–7.5%/yr) during the follow-up, consisting of intracerebral hemorrhage in 6 patients (2.6, 95% CI 0.98–5.8%/yr) and recurrent ischemic stroke in 3 patients (1.3, 95% CI 0.27–3.8%/yr). In the WF group (n = 36), there were 5 patients (4.4, 95% CI 1.4–10.4%/yr) with intracerebral hemorrhage and 2 patients (1.7, 95% CI 0.21–6.4%/yr) with recurrent ischemic stroke, whereas, there was 1 patient (0.8, 95% CI 0.02–4.7%/yr) with intracerebral hemorrhage and 1 patient (0.8, 95% CI 0.02–4.7%/yr) with recurrent ischemic stroke in the DOAC group (n = 45), showing that intracerebral hemorrhage was more prevalent in the WF group. New lobar CMBs occurred in 4 of the 7 patients in the WF group with subsequent stroke, whereas there were no new CMBs in the 2 patients in the DOAC group with subsequent stroke. The presence of ≥ 2 CMBs at baseline was associated with a higher risk of subsequent stroke (OR 7.25, 95% CI 1.01–52.35, P = 0.048). Kaplan-Meier survival curves showed no statistically significant differences in cumulative risk during the follow-up period between the WF group and the DOAC group (log-rank test: P = 0.123 for subsequent stroke). **(S1 Fig)**.

## Discussion

This prospective observational cohort study demonstrated that the development of new CMBs and intracerebral hemorrhage was less frequent among patients prescribed DOACs compared to WF. Regarding location, new CMBs associated with OAC use were mainly lobar lesions. In cardioembolic stroke patients with atrial fibrillation, there has been little prospective research on the association between OACs and the incidence of CMBs, therefore, the present study provides new information on this relationship.

In Japan, the number of elderly cardioembolic stroke patients increases every year, so the prevention of stroke onset and recurrence using OACs is important for reducing medical expenditures. Long-term oral anticoagulation with WF and DOACs is highly effective for ischemic stroke prevention in cardioembolic stroke patients. However,　devastating symptomatic intracerebral hemorrhage is an especially serious complication for Asian patients who are prone to bleeding. Several observational studies and meta-analyses [19–21] suggest a link between CMBs on MRI and increased future intracerebral hemorrhage risk in patients with atrial fibrillation. The CROMIS-2 study, a multicenter prospective observational cohort study, that enrolled patients with atrial fibrillation and recent stroke or TIA treated with WF or DOACs, showed that the presence of CMBs was independently associated with symptomatic intracerebral hemorrhage [20]. A recently reported pooled analysis of the association between CMBs and stroke risk showed that the absolute risk of ischemic stroke was higher than that of intracerebral hemorrhage [9]. As reasons for the discrepancies with our results, the sample size in the present study was small, and there was also the problem of selection bias. In addition, the PT-INR in the WF group was rigorously controlled, suggesting that there were few cases of recurrent ischemic stroke. To date, there have been very few longitudinal studies on the association between anticoagulant use and CMB incidence. If DOACs reduce the development of CMBs, they might cause less intracerebral hemorrhage than WF. In the longitudinal study of Saito et al [22], which followed 69 patients with nonvalvular atrial fibrillation (9 with stroke history) for a year, CMBs occurred in 9 patients (13%), and they were the lobar type in 6 patients. No CMBs occurred in those using DOACs. In our study on secondary prevention in stroke patients with atrial fibrillation during the 3-year follow-up period, the background factors differed from those in this previous study, but, common to both, lobar CMBs were frequent in patients who received WF, and DOAC use did not readily influence CMB appearance. In this context, prospective research conducted in China [23] reported that the duration of DOAC exposure was not associated with the prevalence or burden of CMBs in patients with atrial fibrillation. Similarly, in our study, there was no association between the duration of OAC use and the development of new CMBs. Additionally, a recent multicenter prospective observational cohort study [24] found that, in stroke patients with atrial fibrillation and 1 or more CMBs, the rate of patients with an increase in CMBs tended to be lower in a DOAC group than in a WF group, but there was no difference in the locations where the CMBs appeared. As reasons for the differences with the results of our research, factors such as a difference in follow-up period, lower blood pressures and prevalence of hypertension at baseline may have had an influence. Dabigatran did not increase the incidence of CMBs in either study. In the future, it will be necessary to conduct a longitudinal study on the associations of different types of DOACs and the development of CMB.

Several randomized controlled trials have demonstrated a higher incidence of intracerebral hemorrhage with WF than with DOACs, and WF has been shown to be more likely to reduce vitamin K-dependent factor VII compared with dabigatran [25].

During the 3-year follow-up period in the present study, intracerebral hemorrhage was more prevalent in patients receiving WF (1 patient also taking aspirin) than in those receiving DOACs (1 patient in DOAC group vs. 5 patients in WF group). However, given the influence of selection bias from leaving the choice of medication to the treating physicians, this result might not necessarily be confirmed by randomized study findings. In addition, brain MRI conducted before the onset of intracerebral hemorrhage revealed that there were no CMBs in the same locations where hemorrhage occurred. Thus, although the development of new CMBs is associated with the risk of hemorrhagic stroke, the resulting increase in CMBs would not suggest increased hemorrhage risk for the same location. Interestingly, regarding location, new CMBs were frequently the lobar type (approximately 80%), and few were the deep or infratentorial type in patients taking OACs in our study. For the deep type, an association with hypertensive vasculopathy is conceivable, and a strong association with blood pressure has been suggested. In the present study, blood pressures at discharge were in an optimal range and strictly controlled during the follow-up period, so it was likely that few deep CMBs would have developed. Furthermore, several studies have also reported an association of CAA and CMBs, and studies performed in Western countries have found that lobar CMBs are frequent in CAA patients, suggesting that this association is a factor in the high incidence of subcortical hemorrhage in patients taking WF [26,27]. Since the pathophysiology of CAA may be involved in patients with atrial fibrillation who have small cortical infarcts and numerous lobar CMBs, anticoagulation with DOACs should be considered [28].

Several limitations of the present study should be addressed. First, it was a single-center prospective observational study with a small number of patients. In addition, all patients were taking OACs and there was no control group for comparison, making it difficult to state that there was a causal relationship between OACs and CMB development. However, there were no major differences in the background factors between the DOAC group and the WF group, and the mean follow-up was 3 years in both groups, so we believe that there is no problem with the results of the analysis. Second, the results of the present study may have been affected by selection bias because the patients enrolled were those capable of visiting the hospital as outpatients, so there were no serious cases, and there was a high drop-out rate during the follow-up period. Third, leaving the choice of agent to the discretion of the treating physicians was the cause of variation in the DOAC used. This also resulted in WF being used rather than DOAC for some patients. Factors that led to choosing warfarin instead of DOAC were not fully evaluated and the interaction of such factors could confound the comparison. Fourth, a few patients were taking antiplatelet agents as well as OACs, but as there was no significant difference in CMB development between the 2 groups, this was not thought to have affected the results. Finally, T2*-weighted MRI was used for the evaluation of CMBs in all patients and it is possible that the results would have been different if evaluations had been carried out with susceptibility weighted imaging, which has higher detection sensitivity. Any CMBs smaller than 2 mm that were missed on baseline T2*-weighted MRI might have been misidentified as new CMBs at follow-up.

In conclusion, DOAC use at discharge was associated with a lower incidence of new CMBs as compared with WF in cardioembolic stroke patients with atrial fibrillation. The hypothesis that DOACs are associated with lower rates of development of new CMBs requires testing in a prospective randomized clinical trial.

## Supporting information

S1 Fig Kaplan-Meier curves for the incidence of subsequence stroke during follow-up period in cardioembolic stroke patients with oral anticoagulants (OACs). (PPTX)

S1 Dataset (XLSX)
