# Supplementary material for: Oral anticoagulant use and the development of new cerebral microbleeds in cardioembolic stroke patients with atrial fibrillation
Source: PLoS One. 2020 Sep 17;15(9):e0238456. doi: 10.1371/journal.pone.0238456 (PMC7498025; doi:10.1371/journal.pone.0238456)
Supplement: S1 Fig — (PPTX) [file pone.0238456.s001.pptx]

## Slide 1
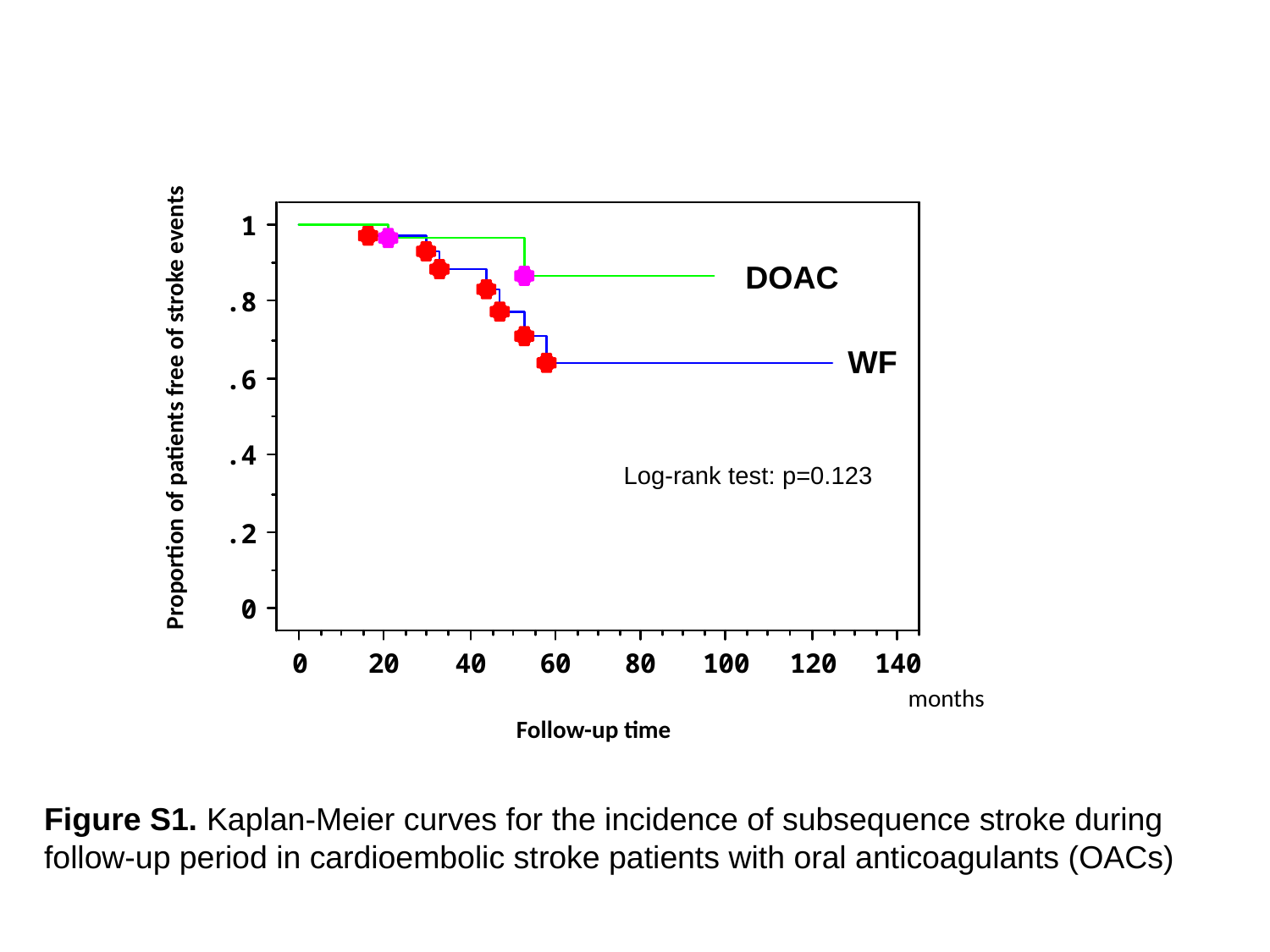

DOAC
WF
Proportion of patients free of stroke events
Log-rank test: p=0.123
months
Follow-up time
Figure S1. Kaplan-Meier curves for the incidence of subsequence stroke during
follow-up period in cardioembolic stroke patients with oral anticoagulants (OACs)
